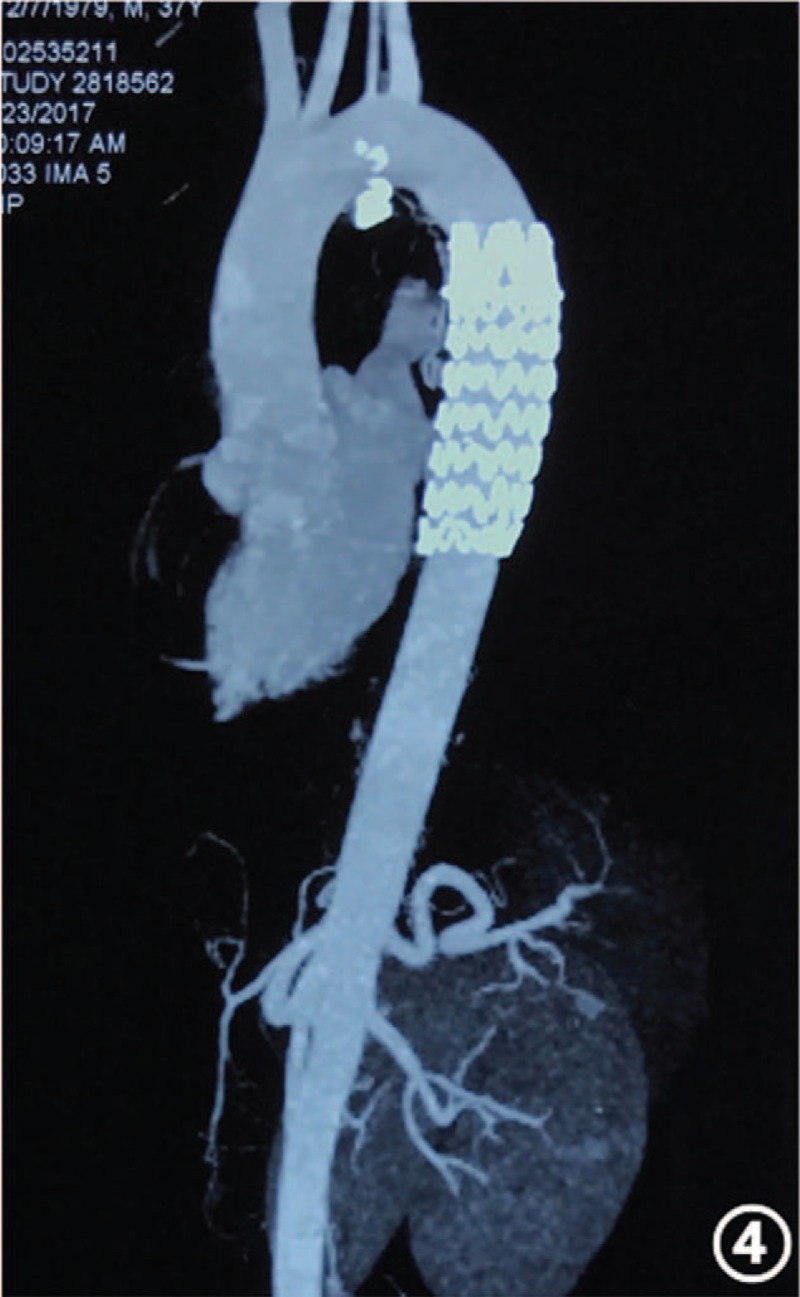# Endovascular treatment of pulmonary sequestration with thoracic endograft: Two case reports: Erratum

**DOI:** 10.1097/MD.0000000000017603

**Published:** 2019-10-11

In the article, “Endovascular treatment of pulmonary sequestration with thoracic endograft: Two case reports”,^[1]^ which appears in Volume 98, Issue 31 of *Medicine*, Figures 1 and 4 appear incorrectly and should be:

Figure 1

**Figure:**
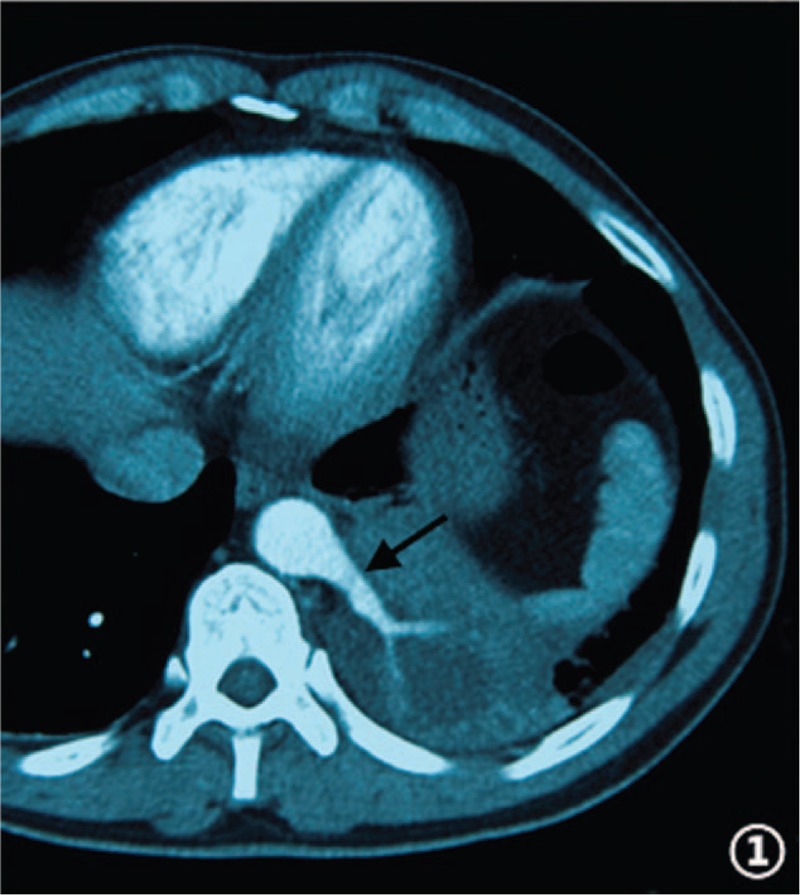


Figure 4

**Figure:**